# Development and clinical validation of a non-invasive, beat-to-beat blood pressure monitoring device, compared to invasive blood pressure monitoring during coronary angiography

**DOI:** 10.31744/einstein_journal/2019AO4156

**Published:** 2019-03-08

**Authors:** Melania Aparecida Borges, Marcelo Prado, Tales Roberto de Souza Santini, Adriano Henrique Pereira Barbosa, Antonio Carlos Moreira, Eder Issao Ishibe, Marcelo Katz, Fatima Dumas Cintra

**Affiliations:** 1 Escola Paulista de Medicina, Universidade Federal de São Paulo, São Paulo, SP, Brazil.; 2 Universidade de São Paulo, São Carlos, SP, Brazil.; 3 University of Pittsburgh, Pittsburgh, PA, United States.; 4 Universidade Federal de São Paulo, São Paulo, SP, Brazil.; 5 Hospital Israelita Albert Einstein, São Paulo, SP, Brazil.

**Keywords:** Coronary angiography, Blood pressure, Validation, Monitoring, physiologic, Angiografia coronária, Pressão sanguínea, Validação, Monitorização fisiológica

## Abstract

**Objective:**

To develop and test a beat-to-beat blood pressure monitoring device during coronary angiography, and compare it with invasive blood pressure monitoring.

**Methods:**

Twenty-eight patients with an indication for hemodynamic study were selected for this investigation, and kept in supine position. Before starting the coronary angiography, they were instructed about the use of the left radial bracelet for beat-to-beat blood pressure monitoring.

**Results:**

There was a significant difference between the time required for the catheterization laboratory team to acquire the first invasive blood pressure reading and the time to obtain the first beat-to-beat reading (11.1±5.1 and 1.5±1.8, respectively; p<0.0001). The intraclass correlation coefficients (95%CI) of systolic and diastolic blood pressures were 0.897 (0.780-0.952) and 0.876 (0.734-0.942), indicating good reproducibility.

**Conclusion:**

This study showed the process to develop a beat-to-beat blood pressure monitoring device. When compared to invasive blood pressure monitoring, there were no significant differences between the two methods. This technique may play a promising coadjuvant role when combined with invasive monitoring during coronary angiography procedures.

## RESUMO

**Objetivo:**

Desenvolver e validar um equipamento para monitorização de pressão arterial batimento a batimento, durante a realização de coronariografia, e comparar com as medidas de pressão arterial invasiva obtidas.

**Métodos:**

Foram selecionados 28 pacientes com indicação de estudo hemodinâmico, que permaneceram em decúbito dorsal horizontal e, antes do início da coronariografia, foram orientados quanto ao uso da pulseira radial esquerda para monitorização da pressão arterial batimento a batimento.

**Resultados:**

Houve diferença significativa entre o tempo necessário para a equipe de hemodinâmica adquirir a primeira medida da pressão arterial invasiva e o tempo da primeira medida da pressão arterial batimento a batimento (11,1±5,1 e 1,5±1,8, respectivamente; p<0,0001). Os coeficientes de correlação intraclasse (IC95%) da pressão arterial sistólica e da diastólica foram 0,897 (0,780-0,952) e 0,876 (0,734-0,942), indicando boa reprodutibilidade.

**Conclusão:**

Este estudo demonstrou o processo de desenvolvimento de um equipamento para avaliação da pressão arterial batimento a batimento. Quando comparado com a pressão arterial invasiva, não foram encontradas diferenças significativas entre as duas medidas. Essa técnica pode constituir ferramenta coadjuvante promissora, associada à monitorização invasiva, durante procedimentos de coronariografia.

## INTRODUCTION

Blood pressure (BP) variations can occur in short time intervals, secondary to cardiopulmonary reflexes,^(^ ^1^ ^)^ neurohormonal,^(^ ^2^ ^)^ sleep-related^(^ ^3^ ^)^ and even behavioral factors.^(^ ^4^ ^)^ Currently, there is a lot of interest around non-manual BP monitoring methods, since automating this process can contribute to optimizing staff time, and offer greater comfort to patients. In addition, BP readings acquired using automatic methods are comparable to manual readings.^(^ ^1^ ^,^ ^2^ ^)^

On the other hand, in some clinical situations, small variations in BP may occur suddenly and have great relevance, such as in vasovagal reflex, bleedings and cardiac arrhythmias, among others. Most automatic devices have been validated for focal measurements and, very often, there is a delay in the documentation of rapid BP variations. In Brazil, there are no devices for beat-to-beat peripheral arterial tonometry that have been tested and validated for clinical use; therefore, continuous BP monitoring can contribute to the follow-up of these patients.^(^ ^4^ ^)^

Arterial tonometry is performed by a transducer positioned on the radial artery, detecting the pulse wave and calculating systolic and diastolic BP, updating values at every heart beat, continuously and non-invasively.^(^ ^5^ ^)^

During coronary angiography procedures, invasive BP monitoring is mandatory for early detection of hemodynamic changes and complications of the procedure. Beat-to-beat BP monitoring devices with an accuracy comparable to that of invasive BP monitoring may contribute to hemodynamic monitoring during invasive and non-invasive procedures.

## OBJECTIVE

To develop and validate a beat-to-beat blood pressure monitoring device to be used during coronary angiography, and compare its readings to those obtained by invasive blood pressure monitoring.

## METHODS

### Study population

We selected 28 consecutive patients with ordered hemodynamic studies, coming from secondary care services, according to the capacity planning center of the Interventional Cardiology Department of a teaching hospital in the city of São Paulo (SP), in the period between February and September 2016. We enrolled patients of both sexes, aged over 18 years, with an indication for elective coronary angiography due to suspected coronary artery disease. Patients with body mass index (BMI) above 40kg/m^2^, history of thrombosis of the upper limbs (UL), previous amputation of the left upper limb (LUL), significant skin abnormalities, asymmetrical pulses, arrhythmias, emergency procedures, and those with hemodynamic instability were excluded.

### Development of the device

#### Prototype

**Figure 1:**
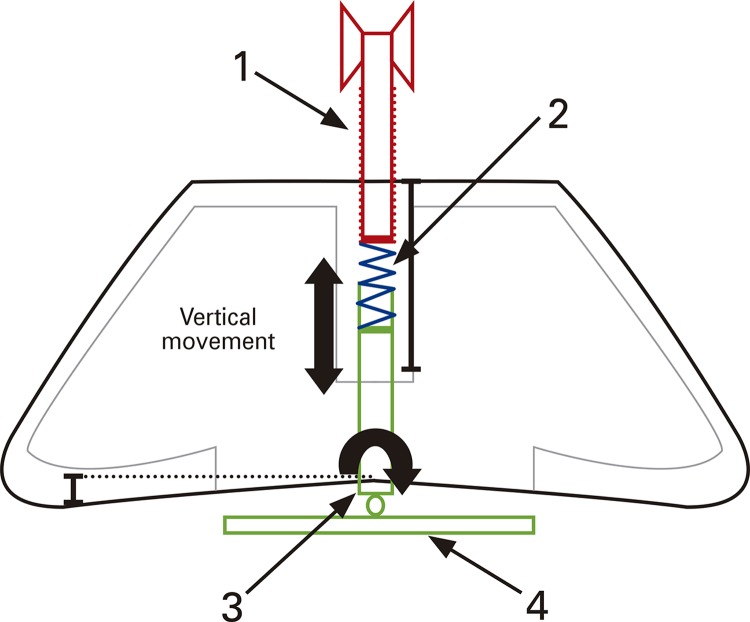
Conceptual model of the bracelet

Three sessions were held to discuss the requirements and develop the prototype in partnership with clinical engineering researchers of the Technological Innovation Center, at *Hospital Israelita Albert Einstein* , based on the principles of peripheral arterial tonometry adapted for the left radial artery. At this stage, the conceptual project of the bracelet was developed ( Figure 1 ).

(1) vertical-adjustment screw; (2) low-stiffness helical spring; (3) three-directional rotation; (4) base for fixation of sensors.

#### Enhancement of the left radial bracelet

At this stage, pre-tests were conducted to verify the feasibility of the measurements and any discomfort when wearing the bracelet. Problems related with ergonomic factors, physical aspects and variations resulting from changing positions were recognized and corrected at this time.

#### Software development

The presentation of data, including heart rate and systolic and diastolic blood pressure values, was defined to ensure easy visualization.

#### Familiarization sessions

Meetings were held with the team to provide the pieces of information they needed, and train them on the proper use of the device, and to start the clinical testing stage.

All stages were conducted in the presence of the medical, nursing and clinical engineering staff.

## Clinical tests

### Clinical trial protocol

Study subjects were instructed to come to the Interventional Cardiology Department with a companion and after fasting for 8 hours. Patients were kept in supine position; before the coronary angiography, they were instructed on the use of the left radial bracelet for beat-to-beat BP monitoring. The bracelet was put on according to the greatest signal strength obtained for the radial pulse. Patients were instructed not to move their left upper limb during the procedure.

After puncturing the femoral or right radial artery and verifying invasive BP using a pressure transducer (TEB SP12 – SP12/32, serial number: 06081004), we calibrated the peripheral arterial tonometry system.

Continuous BP and heart rate monitoring was kept throughout the procedure, even during contrast injection. Blood pressure measurements were collected at six timepoints, during the coronary angiography, at regular intervals, and the data were compared with simultaneous measurements obtained by the invasive system.

## Statistical analysis

We used the software Statistical Package for the Social Science (SPSS), version 20.0, and Stata^®^, for data analysis. The reproducibility between the two measurements was assessed by the intraclass correlation. To facilitate visualization of the two measurements, we plotted Bland-Altman charts. Additionally, we presented Pearson correlations to assess the linear association between the two results. Data were expressed as mean and standard deviation for quantitative variables. Categorical variables were presented as percentages. To compare the means of the two measurements, we used the Student’s *t* test for paired samples. Linear regression models were adjusted to verify the existence of any interaction of the systolic pressure measured with the device and BMI, diabetes or hypertension, with the systolic pressure measured by the invasive method. The same was done for diastolic BP. Both the Student’s *t* test and the linear regression assumed a normal distribution of the data, which was verified by the Kolmogorov-Smirnov test. The significance level was set at p<0.05.

## Ethical aspects

The study was approved by the Institutional Review Board of the *Universidade Federal de São Paulo* (UNIFESP) under opinion 534.636, CAAE: 15481413.0.0000.5505, and all patients signed an Informed Consent Form.

## RESULTS

**Figure 2:**
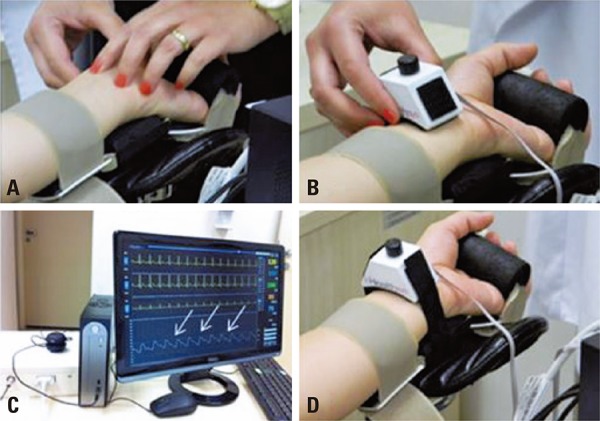
System for positioning of the arterial tonometry bracelet on the left arm and palpation of the left radial pulse

The prototype development and bracelet enhancement stages are shown in figure 2 .

The mean age of patients was 61.5±9.9 years. Of the 28 subjects, 17 were males. The characteristics of the sample investigated are presented in table 1 . There was a significant difference between the time required for the catheterization laboratory (cath lab) team to obtain the first invasive BP reading and the first beat-to-beat BP reading (11.1±5.1 minutes *versus* 1.5±1.8 minutes; p<0.0001). During clinical tests, one patient (0.3%) asked for the bracelet to be removed due to pain on the left wrist. Continuous, beat-to-beat BP measurement was not lost in any of the patients during monitoring, even during contrast injection for coronary angiography.

**Table 1:** Characteristics of the sample investigated

Age ranges	
Age, years	61.5±9.9
Male	60.7
Diabetes	35.7
Hypertension	57.1
Dyslipidemia	28.5
Delta T for invasive*, minutes	11.1±5.1
Delta T for the device^†^, minutes	1.5±1.6

Data expressed as mean±standard deviation, or %. * time required for the cath lab team to obtain the first invasive blood pressure reading; ^†^ time to obtain the first beat-to-beat blood pressure reading.

There was no statistical difference between mean systolic BP values of the device when compared with invasive monitoring (130.73mmHg±15.95mmHg *versus* 128.79mmHg±16.24mmHg; p=0.305). There was no statistical difference between mean diastolic BP values of the device, when compared with invasive monitoring (78.26mmHg±13.23mmHg *versus* 77.57mmHg±12.54mmHg; p=0.677). The intraclass correlation coefficients (95%CI) of systolic and diastolic BP were 0.897 (0.780-0.952) and 0.876 (0.734-0.942), indicating good reproducibility.

**Figure 3:**
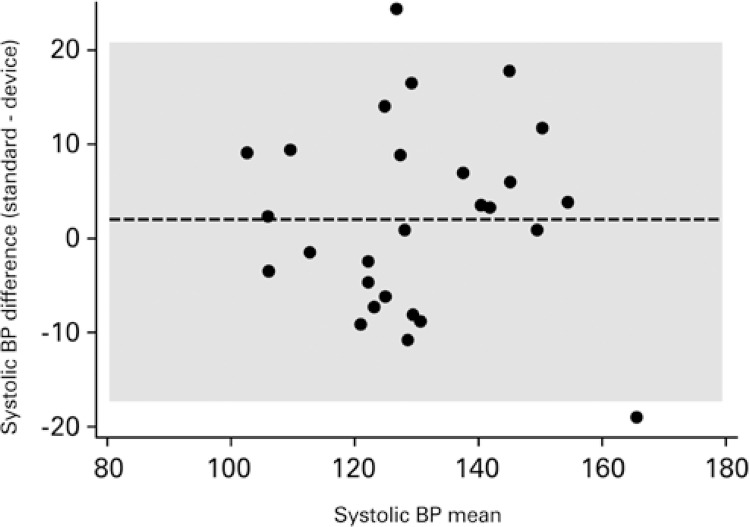
Bland-Altman for systolic blood pressure

**Figure 4:**
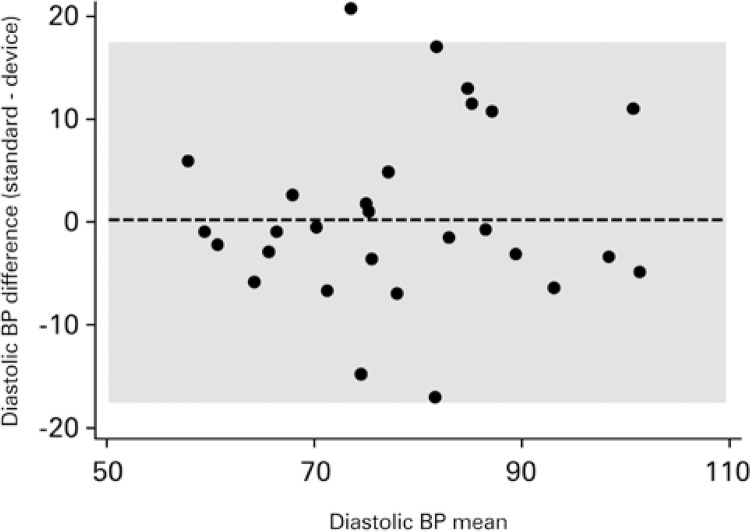
Bland-Altman for diastolic blood pressure

Figures 3 and 4 show the dispersion between readings for systolic and diastolic BP.

BP: blood pressure.

BP: blood pressure.

Linear regression models did not show any interaction effect between BMI, diabetes or hypertension, both for systolic and diastolic BPs measured by the device in relation to the pressure values invasively measured. The beta values of interaction (95%CI) for systolic and diastolic BP and diabetes were, respectively, 1.78 (-0.11- 0.97) and -0.44 (-0.75-0.44). The beta values of interaction (95%CI) for systolic and diastolic BP and hypertension were, respectively, 0.76 (0.24-1.25) and -1.25 (0.98-0.17). The beta values of interaction (95%CI) for systolic and diastolic BP and BMI were, respectively, -1.69 (-0.10-0.21) and -2.93 (-0.18-0.01).

## DISCUSSION

The main finding of this study was the development of a tool capable of monitoring BP beat-to-beat, with good correlation with invasive measurements. It seems to be an international consensus, and also advocated by the Multicenter Meeting on Hypertensive Crises that, once the emergency is confirmed, the situation always requires the use of injectable drugs through proper venous access, if possible using continuous infusion pumps and rigorous BP monitoring.^(^ ^6^ ^)^

The validation of new devices for this purpose is no novelty. Since 1980, several protocols have been proposed, but it was only in 2010 that the European Society of Hypertension published a review with the international protocol for BP measurement in adults,^(^ ^7^ ^)^ which guided many clinical trials concerning technological development and validation protocols. Heusdens et al., investigated 25 consecutive patients undergoing endarterectomy and requiring BP monitoring, and compared non-invasive readings taken with a finger monitor with invasive BP readings, demonstrating a good clinical correlation.^(^ ^8^ ^)^ Hellman et al., looked into patients with Parkinson’s disease, with and without documented orthostatic hypotension, subjected to the Valsava maneuver using traditional methods and continuous, beat-to-beat BP monitoring. The authors demonstrated that continuous monitoring is more sensitive than traditional monitoring for diagnosis of postural hypotension in this population.^(^ ^9^ ^)^ Similar findings were reported by Langwieser et al., who studied the role of peripheral arterial tonometry in patients admitted to intensive care units, demonstrating that the technique has good accuracy when compared to invasive techniques, and can serve as an alternative for monitoring these patients.^(^ ^10^ ^)^ Our study, likewise, showed no significant differences between systolic and diastolic BP readings during coronary angiography.

On the other hand, in a study conducted by Gupta et al., looking at continuous, beat-to-beat monitoring, with intermittent BP measurements during elective cesarean sections, it was demonstrated that patients monitored with the continuous technique had lower detection of BP drops and lower use of oxytocin when compared with the group on intermittent monitoring. The authors concluded that continuous monitoring can only be used as a support to conventional monitoring.^(^ ^11^ ^)^

Furthermore, the concern with the safe use of devices is nationally reinforced in the Patient Safety Program, and actually is one of the program’s goals; for this reason, tolerance to the method was also an important factor investigated in this study, where only one (3.6%) of patients reported local pain with the use of the bracelet.^(^ ^12^ ^)^

The total monitoring time may also be a determinant factor when assessing tolerance and, indeed, in our study, the beat-to-beat monitoring time was approximately one hour, which makes it impossible to compare with monitoring methods with different duration.

The time of acquisition of the first BP reading by arterial tonometry was shorter when compared with invasive pressure monitoring (11.1±5.1 minutes *versus* 1.5±1.8 minutes; p<0.0001). This fact can be important in more urgent scenarios and is a promising finding for future clinical investigations. Another advantage is the fact that beat-to-beat BP monitoring remained unchanged during contrast injection, which can yield additional information during the procedure.

Also, beat-to-beat BP control can be useful in post-cardiac arrest myocardial dysfunction and in the control of hypertensive crises, since the drop in ejection fraction and increased end-diastolic pressure of the left ventricle can take place hours later, with hypotension and low cardiac output and,^(^ ^13^ ^)^ therefore, this control PA must be rigorous.^(^ ^6^ ^)^

Some limitations are worth noting: the small number of patients and the exclusion of obese patients, patients with cardiac arrhythmias and hemodynamic instability, which warrants the need for new studies. Moreover, reproducing these results in other areas is essential for understanding their usability, once the population investigated in this study was quite specific.

## CONCLUSION

There was a correlation between the blood pressure readings using the invasive technique and the non-invasive, beat-to-beat blood pressure monitoring device. Also, greater agility was observed in obtaining the first blood pressure reading. Therefore, the device developed may become a promising tool for hemodynamic monitoring.
